# Implementing the time-to-event continual reassessment method in the presence of partial orders in a phase I head and neck cancer trial

**DOI:** 10.1186/s12874-024-02142-4

**Published:** 2024-01-13

**Authors:** Amit Patel, Kristian Brock, Daniel Slade, Claire Gaunt, Anthony Kong, Hisham Mehanna, Lucinda Billingham, Piers Gaunt

**Affiliations:** 1 Cancer Research Clinical Trials Unit, University of Birmingham, Birmingham, UK; 2 Oncology R&D, AstraZeneca, Cambridge, UK; 3 Department of Oncology, King’s College London, London, UK; 4 Institute of Head and Neck Studies and Education, University of Birmingham, Birmingham, UK

**Keywords:** PO-TITE-CRM, ADePT-DDR, Toxicity, Dose-finding, Partial orders, Monotonicity assumption, Phase I trial

## Abstract

**Background:**

In this article we describe the methodology of the time-to-event continual reassessment method in the presence of partial orders (PO-TITE-CRM) and the process of implementing this trial design into a phase I trial in head and neck cancer called ADePT-DDR. The ADePT-DDR trial aims to find the maximum tolerated dose of an ATR inhibitor given in conjunction with radiotherapy in patients with head and neck squamous cell carcinoma.

**Methods:**

The PO-TITE-CRM is a phase I trial design that builds upon the time-to-event continual reassessment method (TITE-CRM) to allow for the presence of partial ordering of doses. Partial orders occur in the case where the monotonicity assumption does not hold and the ordering of doses in terms of toxicity is not fully known.

**Results:**

We arrived at a parameterisation of the design which performed well over a range of scenarios. Results from simulations were used iteratively to determine the best parameterisation of the design and we present the final set of simulations. We provide details on the methodology as well as insight into how it is applied to the trial.

**Conclusions:**

Whilst being a very efficient design we highlight some of the difficulties and challenges that come with implementing such a design. As the issue of partial ordering may become more frequent due to the increasing investigations of combination therapies we believe this account will be beneficial to those wishing to implement a design with partial orders.

**Trial registration:**

ADePT-DDR was added to the European Clinical Trials Database (EudraCT number: 2020-001034-35) on 2020-08-07.

## Introduction

Typically the main aim of a phase I dose-finding trials is to identify the maximum tolerated dose (MTD) of the treatment being investigated. The MTD is usually determined under the monotonicity assumption which assumes that as dose increases so does the probability of toxicity. With model-based designs such as the continual reassessment method (CRM) escalation occurs to identify the dose with an associated probability of toxicity based on a pre-defined target.

The investigation of multiple-agent treatments in phase I dose-finding trials, where the monotonicity assumption in relation to the dose-toxicity model may not hold, is increasing in early phase trials. Finding the MTD in combinations of treatments, compared to single-agents, presents methodological challenges. Each drug individually may obey the monotonicity assumption we can refer to this as the doses being fully ordered. However, when multiple treatments are combined, the ordering of doses in terms of toxicity may not be fully apparent or may only be partially ordered. An order may be identified for a subset of the doses which would result in a partial order. Without a fully understood ordering it is uncertain which dose should be chosen in decisions of escalation and de-escalation and ultimately as the MTD. This issue is not exclusively reserved for trials with multiple-agents. The monotonicity assumption may not hold for certain drugs in single-agent studies leading to partial orders of dose toxicity. For example, when dose and frequency of administration vary between dose levels. Monotonicity is a very strong assumption. It requires that the probability of toxicity is always increasing - staying the same is not enough. At high enough doses, this assumption is almost surely violated for all interventions when the event probability reaches its maximum. Thus, even when total ordering is possible, the monotonicity assumption could be violated [1]. This can occur in scenarios where multiple parameters of the treatment schedule are altered for each dose level. For example, two doses could prescribe the same overall total dose but be over different treatment durations and hence have higher and lower daily doses. In this situation, it could be unclear as to whether prolonged exposure to a lower daily dose is more toxic than short exposure to a higher daily dose, which implies a partial ordering of toxicity probabilities. This is the case for the proposed dose levels in the ADePT-DDR trial.

Worldwide there are approximately 600,000 new cases of Head and Neck Squamous Cell Carcinoma (HNSCC) each year [2]. Of which, 12,000 occur in the UK with the most common forms of treatment being surgery, radiotherapy and/or chemotherapy. Radiotherapy is essential for the treatment of cancer. It has been estimated that more than 40% of patients will receive radiotherapy at some point in their treatment [3]. However, despite recent advancements in radiation techniques and the use of concomitant chemoradiotherapy, patients with solid tumours such as head and neck cancer have suboptimal cure rates [4]. For those with advanced HNSCC, primary radiotherapy with concurrent chemotherapy is often offered but, it has not been shown to improve survival in patients aged over 70 compared to radiotherapy alone [5]. Therefore, any strategy to improve the efficacy of radiotherapy without increasing toxicity would have a significant impact on patient outcomes. DNA damage repair (DDR) inhibition is a potential technique which could be utilised as it potentiates the therapeutic effects of ionising radiation in cancer cells [6]. Combining radiotherapy with DDR inhibition could improve clinical outcomes for these patients [7].

The ADePT-DDR trial is a platform trial which aims to evaluate the safety and efficacy of different DDR agents, or different immunotherapy agents and/or DDR and immunotherapy combinations, together with radiotherapy in patients with HNSCC. The initial component of this trial is a single-arm dose-finding trial investigating the ataxia telangiectasis and Rad3-related (ATR) inhibitor AZD6738 in combination with radiotherapy. ATR inhibitors not only stop DNA repair but impair the mechanism that allows for repairs to take place. Preclinical models have shown this double blocking to be effective in killing cancer cells [8]. The aim of this trial is to determine a maximum tolerated dose of AZD6738 in combination with radiotherapy.

Further methodological challenges revolve around the issue of late-onset toxicities. Typically, early phase trials implement a short window to observe DLTs (Dose Limiting Toxicities). This works well in situations where toxicities are likely to occur rapidly after treatment. However, this is not optimal for treatments that could cause late-onset toxicities such as radiotherapy. The aim with ADePT-DDR would be to incorporate a larger observation window to account for potential late-onset toxicities from radiotherapy whilst also minimising the trial duration.

Due to the historical use of rule-based designs, the majority of the terminology used to describe them, and the ambiguity they raise, have been inherited by modern designs such as the CRM. The MTD in the context of a CRM is not the ‘maximum’ dose patients could tolerate but rather a dose in which there would be an acceptable target probability of a DLT occurring. For example, if the target is set at 25% the MTD would be the dose at which there is a 25% probability of experiencing a DLT. Rather than using the term MTD, the dose to be found will be referred to as the target dose (TD%%, where the %’s are replaced by the target probability), i.e. TD25 would be the dose expected to be toxic in 25% of patients. We will use this terminology throughout the paper.

The continual reassessment method for partial orders (PO-CRM) developed by Wages et al. [9] extends the CRM design by relaxing the assumption of monotonicity and by modelling different potential orders. Wages et al. [9, 10] further developed their work on the PO-CRM to deal with late-onset toxicities by implementing a TITE component. This trial design, referred to as the time-to-event continual reassessment method in the presence of partial orders (PO-TITE-CRM) by the authors, was chosen to be used in ADePT-DDR. We aim to provide insight into the methodology of PO-TITE-CRM through application in a real-world scenario.

## Methods

### The PO-TITE-CRM design

Wages et al. [10] introduced the PO-TITE-CRM design which builds directly upon the PO-CRM design by incorporating a TITE component into the dose-toxicity model. The aim of which is to determine the target dose for combinations of drugs where the monotonicity assumption does not hold, in a setting where late-onset toxicities are possible.

Using the notation of Wages et al. [9, 10], let *M* denote the number of possible orders and *Y* be an indicator of a DLT event. Then for a trial investigating *k* combinations, $d_{1}$,...,$d_{k}$, the dose for the *j*th patient, $X_{j}$, *j* = 1,...,*n* can be thought of as random $x_{j} \in (d_{1}, ..., d_{k})$. For a specific ordering *m*, $m = 1,...,M$ the toxicity probability $R(d_{i})$ is modelled by

1 $$\begin{aligned} R(d_{i}) = \phi _m(d_i,w,\beta ) = w\psi _m(d_i,\beta ) \; i = 1, ..., k; \; m = 1, ...,M \end{aligned}$$

for a weighted dose response model $\phi _m(d_i,w,\beta )$ where $\beta \in (-\infty , \infty )$ is the model parameter of the working dose toxicity model. The weight, *w* as defined by Cheung and Chappel [11], is a function of the time-to-event of each patient and is incorporated linearly within the dose-toxicity model $\psi$ so that $0 \le w \le 1$. Each patient is followed for a fixed amount of time *T*. Let $U_j$ represent the time-to-toxicity of patient *j*. Then for $u \le T$,

2 $$\begin{aligned} P(U_j \le u ) = P(U_j \le u |U_j \le T)P(U_j \le T) \equiv w(u;T) \psi _m(d_i,\beta ). \end{aligned}$$

For simplicity we will refer to the weight function *w*(*u*; *T*) as *w*. The weight function will have to be decided upon by the trials team, dependent on the scenario, a simple linear function or a more complex adaptive weights function could be utilised. There are also several working dose toxicity models which could be used for $\psi$. Wages et al. [9, 10] present their design with the power parameter model given by

3 $$\begin{aligned} \psi _m(d_i,\beta ) = \alpha _{mi}^{exp(\beta )} \; i = 1,...,k; \; m = 1,\ldots ,M. \end{aligned}$$

Here $0< \alpha _{m1}< ...< \alpha _{mk} < 1$ are the prior estimates of DLT probabilities, or skeleton, for each potential ordering. Furthermore, prior probabilities are assigned to each order *M* to account for any prior information regarding the plausibility of each model such that, $p(m) = \{p(1),...,p(M)\}$, where $p(m) \ge 0$ and $\sum _mp(m)=1$. When all orders are equally likely or there is no prior information available on possible orderings the prior is discretely uniform and would be $p(m) = 1/M$.

A Bayesian framework is used and a prior probability distribution $g(\beta )$ is assigned to the parameter $\beta$. The ordering with the largest prior probability is selected as the starting ordering, in the scenario where all priors are equal an ordering is selected at random, subsequently a starting dose is also chosen. After *j* patients have been entered into the trial, data is collected in the form of $\Omega _j = \{x_1,y_1, ..., x_j,y_j\}$. A weighted likelihood for the parameter $\beta$ is used to establish running probabilities of toxicity for each treatment combination. The weighted likelihood under ordering *m*, is given by

4 $$\begin{aligned} \tilde{L}_m(\beta |\Omega _j)=\prod _{l=1}^{j}\phi _m^{y_l}(x_l,w_l,\beta )\{1-\phi _m(x_l,w_l,\beta )\}^{(1-y_l)} \end{aligned}$$

which can be used to generate a summary value $\hat{\beta }_{mj}$ for each ordering. With the likelihood and the data $\Omega _j$, the posterior density for $\beta$ can be calculated using

5 $$\begin{aligned} \tilde{f}_m(\beta |\Omega _j)=\frac{\tilde{L}_m(\beta |\Omega _j)g(\beta )}{\int _{\beta }\tilde{L}_m(\beta |\Omega _j)g(\beta )d\beta } \end{aligned}$$

This can then be used to establish posterior probabilities of the orderings given the data as

6 $$\begin{aligned} \tilde{\pi }(m|\Omega _j)=\frac{p(m)\int _{\beta }\tilde{L}_m(\beta |\Omega _j)g(\beta )d\beta }{\sum _{m=1}^{M}p(m)\int _{\beta }\tilde{L}_m(\beta |\Omega _j)g(\beta )d\beta }. \end{aligned}$$

We select the single ordering, *h*, with the largest posterior probability along with its associated working model $\psi _h(d_i,\beta )$ and generate toxicity probabilities for each dose level. Once the *j*th patient has been included the posterior probability of DLT can be calculated for $d_{i}$ so that

7 $$\begin{aligned} \hat{R}(d_i) = \psi _h(d_i,\hat{\beta }_{hj}); \; \hat{\beta }_h = \int _{\beta }\beta \tilde{f}_h(\beta |\Omega _j)d\beta . \end{aligned}$$

In turn, the dose level $x_j \in \{d_1,...,d_k\}$ assigned to the ($j+$1)th patient is the dose, $d_i$, which minimises

8 $$\begin{aligned} \triangle (\hat{R}(d_i),\theta ) = |\hat{R}(d_i)-\theta |, \; i=1,...,k \end{aligned}$$

where $\theta$ is the target DLT rate. Similarly, once all patients have been recruited and observed and the trial ends, the target dose (TD$\theta$) is the dose, $d_{i}$, which minimises (8).

### PO-TITE-CRM in ADePT-DDR

The intended use of this design is for dose-finding in combinations of therapies, as this is the main source of the partial ordering issue. ADePT-DDR however, is a unique implementation of the design as, even though it involves a combination of therapies (radiotherapy and AZD6738), the dose of radiotherapy is fixed and dose-finding is only planned for AZD6738. PO-TITE-CRM is still applicable in this case as the design includes combinations of dose and duration for AZD6738 which are partially ordered. A summary of the proposed dose levels can be found in Table 1.

**Table 1:** ADePT-DDR dose-levels and duration of treatment for AZD6738

Dose Level	AZD6738 Daily dose (mg BD)	Weeks	Duration (days)	Radiotherapy
-1	20	1	5	70 Gy/ 35 F
0	20	1 &4	10	70 Gy/ 35 F
1	40	1 &4	10	70 Gy/ 35 F
2a	40	1,2,4 &5	20	70 Gy/ 35 F
2b	80	1 &4	10	70 Gy/ 35 F
3	120	1 &4	10	70 Gy/ 35 F
	80	1,2,4 &5	20	70 Gy/ 35 F

A two-stage PO-TITE-CRM will be used to find the TD25 of AZD6738. This will be determined by DLTs evaluated by Common Terminology Criteria for Adverse Events (CTCAE) v5.0 and Radiation Therapy Oncology Group (RTOG) late toxicity score. The binary DLT events are pre-defined by a variety of grade 3-4 adverse events notably, haematological, cardiovascular and gastrointestinal/hepatic toxicities as well as significant non-haematological events and specific treatment-related toxicities. DLTs will be monitored for the duration of treatment (seven weeks) and throughout the follow-up period. The total follow-up period post treatment is 52 weeks, so patients will spend a total of 59 weeks in the trial.

A maximum of 60 patients will be recruited for the dose-finding aspect of this trial and up to 20 patients as controls. Controls will be utilised to make comparisons for secondary outcomes such as survival and efficacy. Control patients will only be receiving radiotherapy, the dose of which is fixed at 70Gy/35F (control patients will not be included in any of the dose-finding aspects of the trial). Controls will be recruited in the interim period between the recruitment of the third patient in a cohort and the completion of the minimum follow-up period. Additionally, patients can also be recruited to the control dose if they do not wish to receive AZD6738 whilst the dose-finding cohort is actively recruiting.

The first cohorts of patients will be allocated to dose level 0. The first stage of the design will follow an initial escalation scheme escalating cohorts of three patients to dose level 1, 2a, 2b then 3 if no DLTs occur. If a DLT occurs stage I of the design ends and stage II begins. In stage II cohorts of three patients are assigned to dose levels chosen by the PO-TITE-CRM.

**Fig. 1:**
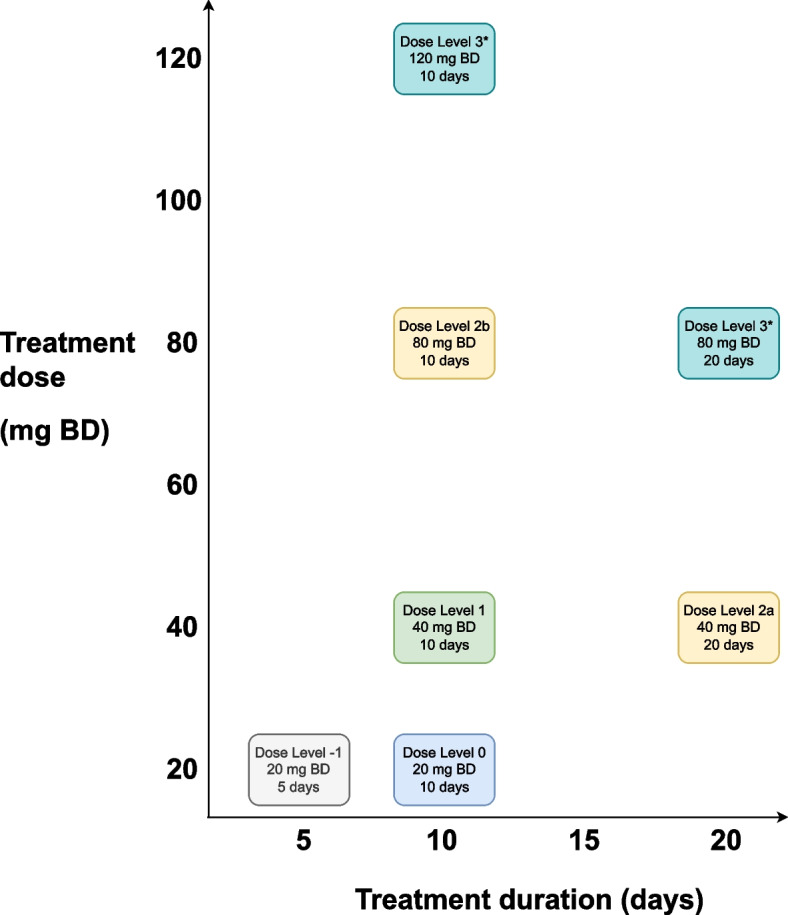
ADePT-DDR dose levels across dose and duration

Each patient entered into ADePT-DDR will receive fixed dose radiation, totalling 70 Gy in 35 fractions over seven weeks. For the dose-finding aspect we investigate six doses of AZD6738 detailed in Table 1. Treatment dose and duration to be selected for dose level 3 will be determined based on a combination of data observed, adverse events and compliance. The issue of partial ordering is illustrated in Fig. 1 inspired from plots by Wages et al. [10]. The doses to be used in this trial are detailed in their appropriate box. Additionally, each dot represents a potential dose combination which theoretically could be investigated. The combinations are colour coordinated to indicate where partial ordering exists in this dose combination space. Doses across the same colour (each diagonal) cannot be distinguished from each other in terms of probability of toxicity. However, it forms a hierarchy in which doses of the same colour can be thought of as less/more toxic that doses in another colour i.e the red dose levels would have a higher probability of toxicity than the yellow dose levels. It is clear that dose levels 2a and 2b would be considered more toxic than dose level 1 due to the increase in treatment duration and treatment dose respectively. However, when comparing 2a and 2b it is unknown whether the increase in dose or duration will be more toxic. Hence there are two possible orderings for ADePT-DDR.

Traditionally, dose-finding trials for combinations would select dose levels to form a ‘path’ through the dose combination space such that each subsequent dose level was logically more toxic. This avoids the issue of partial ordering but means doses of interest or effective dose combinations may be missed or not investigated. Specifically, for ADePT-DDR this allows two ‘paths’ from dose level 1 extending to 2a and 2b. In terms of dose level 3 only one of the doses in that tier will be investigated, it was unclear as to which dose level would be best due to a lack of historical data. The choice of dosing for this dose-level will be determined based on data observed throughout the trial. Even though dose level 3 is not yet specified in terms of modelling and simulations it was treated as singular dose. This was done as clinicians thought that it would be unlikely that we would reach these doses and that the probability of toxicity between them would be similar.

Preliminary designs of the trial included only five dose levels and planned to use dose level 0 as the starting dose. During the trial design phase it was decided a new lower dose (dose level -1) would be introduced to allow for de-escalation if the initial dose was found to be too toxic. Dose escalation/de-escalation for subsequent cohorts would be determined from the two-stage PO-TITE-CRM. A two-stage design allows for escalation according to a pre-defined escalation scheme similar to a ‘3+3’ design. The first stage dictates that if no DLT’s are observed in the current cohort the dose allocated to the next cohort is the following dose in the escalation scheme. Dose levels continue to be incremented in this fashion until the first DLT is observed. In stage two, dose levels are determined by the PO-TITE-CRM.

Typically CRM designs begin by testing the first patient, or cohort, at the prior guess of TD or at a lower dose to be safe. However, clinicians may have safety concerns beginning the trial at higher dose levels as well as escalating to higher dose levels without testing lower ones. Investigators in ADePT-DDR expressed similar concerns as such a two-stage design was adopted. The escalation scheme used in stage one of ADePT-DDR will follow that of the first ordering ($d_{-1} \rightarrow d_{0} \rightarrow d_{1} \rightarrow d_{2a} \rightarrow d_{2b} \rightarrow d_{3}$). If patients in the first cohort (assigned to dose level 0) don’t experience a DLT the next cohort will be allocated to dose level 1 and then if no DLTs are observed again the third cohort will be allocated to dose level 2a and so on and so forth. The dose escalation scheme was determined based on the prior probabilities of toxicity generated for each dose level.

Information elicited from the investigators helped generate prior probabilities of toxicity for each dose level. They believed that dose level 2b would be the TD25 with 2a being less toxic. This was used in conjunction with the getprior function from the dfcrm R package [12] which yielded priors of 0.01, 0.04, 0.08, 0.16, 0.25 and 0.35 for dose levels -1, 0, 1, 2a, 2b and 3 respectively. The half-width of the indifference interval was set at 0.05. The indifference interval is an interval in which the toxicity probability of the selected dose will eventually fall. Prior probabilities are also required for the plausibility of each model and even though the clinicians think that 2b will be more toxic than 2a there is no clear evidence and still a lot of uncertainty. As such it is sensible to assume a plausibility probability of 0.5 for each ordering, implying both orders are equally likely to be the true ordering of these dose levels.

### The TITE component

The observation window for this trial will be up to a year post-treatment as the combination of radiotherapy with AZD6738 is anticipated to cause late-onset toxicity. The acute DLT observation period is 12 weeks (84 days) post radiotherapy end with a minimum of 8 weeks (56 days) for the last patient of each cohort. However, patients will continuously be monitored for occurrence of DLT for at least 12 weeks (84 days), i.e. at least 12 weeks (84 days) from the end of radiotherapy. The full window will last for 52 weeks (365 days) post-treatment.

The TITE component incorporates a weighting contribution for each patient dependent on how long that patient has been evaluable in the study. This allows a patient to be evaluated once they have been observed for the minimum DLT period of 8 weeks (56 days). The weighting at this point is 60% rising to 80% at 12 weeks (84 days). A patient will not contribute fully to the model until they have completed 52 weeks (365 days) follow up (or have experienced a DLT at any stage in which case they will be weighted as a whole contribution). Linear weighting functions will be employed for any patient with a length of follow up between these three time points. One weight function to calculate weights between 8-12 weeks and another for weights between 12-52 weeks. For the weighting function $w(u;t_1, t_2, t_3)$ where *u* is the time-to-toxicity of patient *j* and $t_1, t_2, t_3$ is the time period with values 8, 12 and 52 respectively. Then for $t_1 \le u \le t_3$

9 $$\begin{aligned} w(u;t_1,t_2,t_3) = 0.6 + 0.2\frac{min(0, min(u, t_2) - t_1)}{t_2 - t_1} + 0.2\frac{max(0, u - t_2)}{t_3-t_2}. \end{aligned}$$

**Fig. 2:**
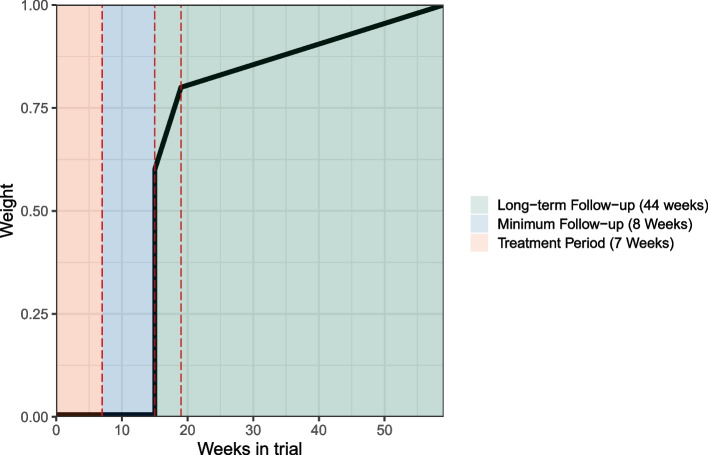
Weights of patients who have not experienced a DLT across the observation window

All patients will have a minimum weight of 60% as that is the prescribed weighting to the minimum follow up period before dose escalation/de-escalation decisions can be made. For each additional week the patient is observed, without a DLT occurring, between weeks 8 and 12 their weighting increases by 5%. Similarly for each week between 12 and 52 weeks, without a DLT, weighting increases by 0.5%. Figure 2 illustrates the weight function and how the weight changes for patients dependent on how long they have been followed-up. The dotted lines represent key time points in the trial. The first being after treatment (7 weeks), the second being the minimum follow-up period at 8 weeks post-treatment (15 weeks into the trial) and the third being at 12 weeks post-treatment (19 weeks into the trial).

The TITE-CRM originally presented by Cheung and Chappel [11] did not incorporate a minimum follow-up period and their design allowed for the continual recruitment of patients whenever they became available. There are some practical considerations which make this infeasible in ADePT-DDR. The model would need to be run each time a new patient entered the study which requires statistical input hence the introduction of cohorts. Clinicians may also have safety concerns if we see rapid recruitment at the start of the trial and the model keeps escalating so we impose a minimum follow-up period. Initially this was set at 12 weeks (at 80% weighting) however, this would have meant that dose escalation/de-escalation decisions would have to take place 19 weeks (7 weeks treatment and 12 weeks follow-up) after recruitment of the third patient in the cohort. Dependent on the recruitment rates this could extend the duration of the trial and negates the benefits of using a TITE design. Consultation with the trial clinicians and the Trial Management Group (TMG) indicated that the trial duration would be too lengthy and settled on lowering this period to 8 weeks (at 60% weighting) whilst also including the original 12 week weighting of 80%.

### Stopping rules

A practical modification was included to allow for early stopping of the trial if there is sufficient evidence that the TD25 has been reached. Sufficient evidence is achieved once 15 patients (five cohorts) have been treated at the same dose level and the model allocates that dose level again to a sixth cohort. This rule evolved from the original designs of the trial which involved 30 patients with a dose expansion cohort to ensure at least 15 patients were treated at the TD25.

Initial simulations highlighted the inadequacy of these design parameters, as operating characteristics for various scenarios were poor, specifically in terms of correct TD25 selection. Clinicians explained the inclusion of the dose expansion cohort was to ensure the dose-finding aspect of the trial did not take a large amount of time whilst also allowing safety to be assessed at the TD25. In order to ensure that a reasonable amount of patients would be treated at the TD25, the trial wouldn’t take longer than necessary and operating characteristics improved, the sample size was increase and this rule was introduced.

A rule was also implemented to allow for early termination of the trial in the case of excess toxicity at the lowest dose. If the probability of DLT at the lowest dose is higher than 0.35 with a probability of 80% and has been tested the trials safety committee will be alerted and will recommend if the trial should be stopped. As the trial starts at dose level 0, which is not the lowest dose, it’s hypothetically possible for the trial to recommend terminating without ever allocating patients to the lowest dose level. As such it was decided early termination would only occur once at least 3 patients (1 cohort) have been allocated dose level -1.

An approximate estimate of the variance was calculated using methodology presented by O’Quigley and Shen [13]. The observed information matrix is obtained by taking the second derivative of the likelihood (eq. 4) which is then used to calculate the variance $v(\hat{\beta _j})$, for estimate $\beta _j$ which becomes more accurate with larger sample sizes. After each cohort, we sample many times from a normal distribution with parameters based on the estimate of $\beta _j$ and its variance. These samples are then plugged into our dose-toxicity model to ascertain the probability of toxicity at the lowest dose. The trial will be recommended to stop if it breaks the rule based on the criteria above.

## Results

Simulations were repeatedly utilised during the design process of the trial to assess how various changes to design features impact the overall performance. Changes to design features such as the sample size, weight function and stopping rules helped inform decisions which led to this design.

Functions from pocrm package in R were modified in order to perform simulations. These modified functions will also be used for analysis during the conduct of the trial. The majority of work involved integrating the TITE component and the stopping rules into the code. In standard CRM designs a binary outcome for toxicity is generated for each patient based on a pre-specified true DLT rates for the dose they are assigned. Adding the TITE component means the time the toxicity occurs also has to be generated, the simulation must also track this time and incorporate this information into the PO-TITE-CRM model when it needs to make dose allocation decisions for the next cohort. We defined multiple scenarios to reflect various real life possibilities in order to assess the designs performance. Simulations presented here were based on the design specified in the previous section, which included six dose levels (-1, 0, 1, 2a, 2b and 3) with dose level 3 treated as a singular dose.

Standard scenarios include adjusting the true DLT rates to reflect each dose being the TD25. For each of these we calculate the probability of selecting each dose as the TD25. It would be expected that the dose with the highest probability of being selected has its true DLT rate set at 25% to match the target rate. A high probability of selecting the correct dose implies the design works well in the specified scenario. Additional characteristics such as the average number of patients at each dose level and how many receive the ideal dose were also investigated. This can be used to look at how many patients may potentially be allocated to a toxic dose. It is also necessary to consider performance when all doses are too toxic, in which case we would want the design to recommend stopping early. Usually the true DLT rates used to define these scenarios abide by the monotonicity assumption. Due to the partial ordering we consider scenarios in which the true DLT rates follow both orders. For trials with a large amount of orders it may be unfeasible to run so many simulations. However, as ADePT-DDR only has two orders we explored all scenarios for each ordering.

We simulated 10000 trials for each scenario using this design detailed in Methods section. It is recommended by Morris et al. [14] to detail the Monte Carlo standard error in order to quantify the simulations uncertainty. The Monte Carlo standard error for probabilities estimated by 10000 simulations is $\sqrt{0.5 \times 0.5/10000} = 0.5\%$. This implies that for any differences in selection probabilities greater than 1% are due to more than simulation error. Simulations were based on the assumption that the trial would recruit one patient per month. The occurrence of DLT’s were randomly generated for patients in each cohort using a Bernoulli distribution with the probability set at the true DLT rate for that cohort’s assigned dose level in the specific scenario. For patients who had a DLT occur, the time at which the DLT occurred was randomly generated using a uniform distribution which spanned the start of treatment to the end of follow-up.

Table 2 details simulations for eight scenarios to test the performance of the PO-TITE-CRM design using true DLT rates which reflect the first ordering. We analyse scenarios where each dose is the TD25 (scenarios 1-6) and when all doses are too toxic (scenario 8). Additionally, we also investigate performance under conditions where the probability of DLT is fairly similar between doses (scenario 7). This is a notoriously difficult circumstance for CRM designs to deal with as the limited number of patients and events at each dose make it hard to accurately estimate toxicity probabilities if they are similar. Simulation results for the second ordering are shown in Table 3 where dose level 2a is considered more toxic than 2b. This is achieved by altering the true DLT rates so 2b has a lower probability of DLT compared to 2a.

**Table 2:** Operating Characteristics of the two-stage PO-TITE-CRM (with true DLT rates that imply 2b is more toxic than 2a) based on 10000 simulated trials. Definitions: DLT: Dose-limiting toxicity. P(select): Probability of selecting a dose as the TD25. Bold values indicate the correct decision

		Dose Levels						
		-1	0	1	2a	2b	3	Stop
Scenario	Prior DLT	0.01	0.04	0.08	0.16	0.25	0.35	
1: TD25 @-1	True DLT rate	0.25	0.4	0.45	0.5	0.55	0.6	
	P(select)	**0.68**	0.18	0.05	0.01	0	0	0.08
	% of patients	39	32	20	6	3	0	
	Mean number of patients	10.17	8.46	5.33	1.67	0.69	0.07	
2: TD25 @0	True DLT rate	0.12	0.25	0.4	0.45	0.5	0.55	
	P(select)	0.23	**0.51**	0.2	0.03	0.02	0	0.01
	% of patients	17	35	29	11	6	1	
	Mean number of patients	5.24	10.48	8.75	3.4	1.83	0.26	
3: TD25 @1	True DLT rate	0.09	0.12	0.25	0.4	0.45	0.5	
	P(select)	0.02	0.2	**0.55**	0.14	0.09	0.01	<0.01
	% of patients	4	20	34	23	16	3	
	Mean number of patients	1.22	6.41	10.97	7.23	5.14	1.02	
4: TD25 @2a	True DLT rate	0.06	0.09	0.12	0.25	0.4	0.45	
	P(select)	0	0.02	0.22	**0.48**	0.23	0.05	<0.01
	% of patients	1	12	20	31	25	11	
	Mean number of patients	0.47	3.88	6.74	10.43	8.2	3.5	
5: TD25 @2b	True DLT rate	0.03	0.06	0.09	0.12	0.25	0.4	
	P(select)	0	0	0.02	0.3	**0.43**	0.25	0
	% of patients	1	10	12	24	28	25	
	Mean number of patients	0.25	3.36	4.15	8.17	9.33	8.33	
6: TD25 @3	True DLT rate	0.01	0.03	0.06	0.09	0.12	0.25	
	P(select)	0	0	0	0.09	0.13	**0.78**	0
	% of patients	0	10	11	18	18	42	
	Mean number of patients	0.1	3.13	3.49	5.46	5.6	13.14	
7: Equal steps in DLT rate	True DLT rate	0.05	0.1	0.15	0.2	0.25	0.3	
	P(select)	0	0.03	0.12	0.31	**0.28**	0.26	<0.01
	% of patients	2	13	18	26	23	19	
	Mean number of patients	0.55	4.03	5.72	8.32	7.15	5.96	
8: All toxic	True DLT rate	0.5	0.6	0.65	0.7	0.75	0.8	
	P(select)	0.26	0	0	0	0	0	**0.74**
	% of patients	56	26	15	2	0	0	
	Mean number of patients	9.05	4.27	2.4	0.37	0.04	0

**Table 3:** Operating Characteristics of the two-stage PO-TITE-CRM (with true DLT rates that imply 2a is more toxic than 2b) based on 10000 simulated trials. Definitions: DLT: Dose-limiting toxicity. P(select): Probability of selecting a dose as the TD25. Bold values indicate the correct decision

		Dose Levels						
		-1	0	1	2a	2b	3	Stop
Scenario	Prior DLT	0.01	0.04	0.08	0.16	0.25	0.35	
9: TD25 @-1	True DLT rate	0.25	0.4	0.45	0.55	0.5	0.6	
	P(select)	**0.67**	0.19	0.05	0	0.01	0	0.08
	% of patients	39	32	20	6	3	0	
	Mean number of patients	10.19	8.43	5.27	1.6	0.68	0.07	
10: TD25 @0	True DLT rate	0.12	0.25	0.4	0.5	0.45	0.55	
	P(select)	0.23	**0.52**	0.2	0.02	0.02	0	0.01
	% of patients	18	36	29	11	6	1	
	Mean number of patients	5.24	10.64	8.82	3.16	1.85	0.24	
11: TD25 @1	True DLT rate	0.09	0.12	0.25	0.45	0.4	0.5	
	P(select)	0.02	0.2	**0.55**	0.09	0.14	0.01	<0.01
	% of patients	4	20	34	21	17	3	
	Mean number of patients	1.16	6.43	11.07	6.83	5.6	1.07	
12: TD25 @2a	True DLT rate	0.06	0.09	0.12	0.25	0.15	0.45	
	P(select)	0	0.01	0.08	**0.44**	0.33	0.14	<0.01
	% of patients	1	11	16	30	24	18	
	Mean number of patients	0.48	3.78	5.24	10.1	7.9	6.07	
13: TD25 @2b	True DLT rate	0.03	0.06	0.09	0.35	0.25	0.4	
	P(select)	0	0	0.15	0.31	**0.43**	0.11	0
	% of patients	1	11	18	30	28	14	
	Mean number of patients	0.25	3.5	5.9	9.82	9.14	4.54	
14: TD25 @3	True DLT rate	0.01	0.03	0.06	0.12	0.09	0.25	
	P(select)	0	0	0	0.13	0.09	**0.78**	0
	% of patients	0	10	11	19	16	43	
	Mean number of patients	0.1	3.13	3.51	5.88	5.06	13.13	
15: Equal steps in DLT rate	True DLT rate	0.05	0.1	0.15	0.25	0.2	0.3	
	P(select)	0	0.02	0.12	**0.32**	0.27	0.26	<0.01
	% of patients	2	13	19	27	22	18	
	Mean number of patients	0.54	4.02	5.93	8.56	6.89	5.75	
16: All toxic	True DLT rate	0.5	0.6	0.65	0.75	0.7	0.8	
	P(select)	0.27	0	0	0	0	0	**0.73**
	% of patients	56	27	15	2	0	0	
	Mean number of patients	9.01	4.28	2.39	0.38	0.05	0	

In scenarios 1 - 6 (Table 2), this design correctly selects the TD25 with probabilities between 43% and 78%, under the assumption 2b is more toxic than 2a. Likewise, for the ordering where 2a is more toxic than 2b, scenarios 9-14 (Table 3) have probabilities between 43% and 78% of correctly selecting the TD25. Correct selection probabilities are generally higher when the TD25 is at the first and last dose levels compared to dose levels 2a and 2b. However, these dose levels are still chosen with the highest probability as the TD25 in their given scenarios. For scenarios 7 and 15, the probabilities of toxicity are equally spaced, approximately 5% apart. This is a relatively diffcult scenario for dose-finding studies to handle. The probability of selecting the TD25 is 28% and 32% for orderings 1 and 2 respectively and even if the performance is poor the correct dose is still likely to be selected. In scenarios 8 and 16, where all the doses are too toxic, the design very seldom allocates patients higher than the first three doses and there is a high chance (74% and 73% respectively) that the trial will recommend early stopping.

Additionally, we assess designs based on the distribution of patients across doses. Designs may correctly select the TD25 however, this could be undesirable and unethical if the majority of patients are over dosed at the more toxic dose levels. The average number and the percentage of patients at each dose level, for each scenario, is recorded in Tables 2 and 3.

The percentage of patients treated at the TD25 ranges between 23% and 43% for each scenario under both orderings. The design also allocates the most patients on average to the TD25 apart from in scenario 7. In this case more patients were allocated to the next lowest dose, we have already discussed the difficulties of this scenario so this characteristic is not too concerning. The mean number of patients recruited for scenarios 1-6 is 26, 30, 32, 33, 34 and 31 respectively. Similarly for scenarios 9-14 its 26, 30, 32, 34, 33 and 31. Even though we allow for up to 60 patients the majority of trials terminate early based on the pre-defined rules for selecting the TD25. This information is presented in Table 4 which also shows how often the max sample size is reached from the 10000 trials for each scenario. We can see in all scenarios, except those where doses are all toxic, we reach the maximum sample size in a small number of simulations. This is largest for scenario 1 where 21 of the 10000 (0.21%) needed the full sample size of 60 patients.

**Table 4:** Summary of simulated patient numbers for each scenario

Scenario	Max no. of patients	% max reached	Mean no. of patients
1: TD25 @-1	60	0.21	26.38
2: TD25 @0	60	0.08	29.97
3: TD25 @1	60	0.05	32.01
4: TD25 @2a	60	0.12	33.22
5: TD25 @2b	60	0.06	33.60
6: TD25 @3	60	0.02	30.92
7: Equal steps	60	0.01	31.74
8: All toxic	54	0.01	16.14
9: TD25 @-1	60	0.17	26.24
10: TD25 @0	60	0.11	29.95
11: TD25 @1	60	0.06	32.15
12: TD25 @2a	60	0.07	33.56
13: TD25 @2b	60	0.03	33.16
14: TD25 @3	60	0.08	30.81
15: Equal steps	60	0.02	31.69
16: All toxic	51	0.01	16.11

Overall, the simulation results show the specification of this design performs relatively well in a number of scenarios. We have shown there is a high probability of the trial stopping early if all dose-levels are too toxic. We have also shown the design behaves in an appropriate manner when there is a lack of disparity between dose-levels in terms of toxicity. Finally, we have demonstrated that regardless of the ordering we observe the PO-TITE-CRM has a high probability of selecting the correct dose. There are a number of limitations to the operating characteristics presented here which are due to the specification of the simulations and trial design.

## Discussion

The PO-CRM and PO-TITE-CRM designs offer solutions to the issue of partial ordering where the order of the doses of treatments are only partially known. The original methodology details that this issue commonly arises in trials of multiple agents, where each drug individually may follow the monotonicity assumption but when combined at certain dose levels this may not hold. This issue is typically dealt with by fixing the dose of one of the agents and escalating the other or escalating both agents simultaneously. This means certain drug combinations that are clinically relevant may not be investigated or even considered.

Here we have shown that these issues can also arise in other situations. Even though the ADePT-DDR trial uses multiple agents the issue of partial ordering occurs due to the varying treatment dose and schedule for one of its agents AZD6738. Implementing the PO-TITE-CRM design allowed us to deal with this issue effectively. There may be other factors or variables in single-agent dose-finding trials that would lead to the issue of partial ordering and would warrant the use of either PO-CRM or PO-TITE-CRM. A limited literature review highlighted that this may be the first instance of the PO-TITE-CRM design being applied. It is important to note that although this methodology takes into account all the various orderings the main aim is to identify the TD%% and does not attempt to identify the order that is more correct.

Compared to other CRM based designs only a few additional pieces of information are required to implement the PO-CRM design, specifically the number of toxicity orderings and prior probabilities for the orders. Dependent on how many dose combinations are available it may not be feasible to investigate all combinations and all orderings. Careful thought and consideration should be given to the combinations and orderings selected which would require input from all relevant investigators (TMG, clinical investigators and other relevant stake holders). In terms of priors for orderings, if no prior information is available all orders should be treated as equally likely to occur. Extending this design to the PO-TITE-CRM requires a fit for purpose weight function and is applied in a similar way to the TITE-CRM methodology. There is an R package available with functions that can be used to run and simulate a PO-CRM trial. These functions were extended to included weighted dose toxicity models as described in this chapter to implement PO-TITE-CRM into ADePT-DDR. The lack of available software for PO-TITE-CRM specifically may be one of the reasons for its lack of use.

In terms of the ADePT-DDR trial, dose combinations were decided upon by the clinical investigators. The issue of partial ordering was due to the dose-levels 2a and 2b and as such this methodology was employed to deal with that scenario. This is a very simple example of partial ordering as we only have two possible orderings and six dose levels. The necessity of implementing this methodology was discussed and whether or not adopting an easier solution by simply altering the dose levels would have been better. Ultimately, the dose levels selected by the clinicians were deemed the most relevant with the TD25 likely to be one of these doses.

Our design used the power model as the working dose-toxicity model. Alternative models such as the one and two parameter logistic model could also be implemented. Whilst a two parameter model may better estimate the dose-toxicity relationship it is unclear if this is still applicable in the presence of partial orders. Therefore, for the purposes of this trial aiming to identify a TD25 a one parameter model was used. As the original authors of the methodology utilised the power model we felt this would be appropriate to use in this trial as well. Further work could be done via simulations to investigate how other models would perform with this design.

Similarly, alternative weight functions such as a polynomial function could also be explored. Our selection of weight function was motivated to a large extent by clinical input. We chose to use a two piecewise linear function due to its simplicity in interpretation. Also, due to the lack of data and certainty around how the weights should actually change over time.

Simulations to generate operating characteristics were the main tools used to assess the designs performance as well as help understand the impact of sample size and stopping rules. This was an iterative process that involved running multiple iterations of simulations under various scenarios until the design was finalised. A key point is that scenarios from simulations should account for each of the possible orderings. ADePT-DDR only has two orderings and we ran scenarios for both. For a trial with a greater number of orderings, this may be unfeasible but at least some scenarios should be assessed to ensure the design is behaving as expected. Overall, the design operating characteristics performed reasonably well even in difficult scenarios.

One limitation of the simulations is how the time-to-event data is generated. The time of DLTs is sampled from a uniform distribution *U*(0, 413), where the time of the DLT can occur at any time between the patient beginning treatment and the end of follow-up (413 days). Using this uniform distribution implies that a DLT has an equal probability of occurring at any time-point in the observation window. This may not be an accurate representation of what happens in the actual trial. Similar comments can be made about the accrual rate used in the simulations. Here we specified the recruitment of one patient per month which is in no way guaranteed for the actual trial. Wages et al. [10], when presenting this methodology investigated four different applications of the PO-TITE-CRM which used different models to enroll patients and allocate DLTs. Results across these four applications were comparable and therefore we assume similar conclusions for this study.

The simulations are also able to instantaneously determine dose-levels for incoming cohorts with all available information. This does not fully reflect the process in which dose-escalation decisions would be made during the actual running of the trial. The analysis would require a data snapshot and time would have to be spent cleaning the data and determining the next dose-level. Meaning any data from the point of the snapshot would not be included in any dose escalation/de-escalation decisions.

## Conclusion

We detail the issue of partial ordering and how we implemented the trial design, in what we believe is the first real-world application of this design. A large amount of simulation work is required to assess the performance of the design. We recommend running several varied scenarios for each potential ordering that will be investigated. This is often an iterative process to refine decisions that were made and often requires input from both clinical and statistical investigators to ensure that the trial design is fit for purpose.

## Data Availability

All data presented in this manuscript is simulated data. The results presented here are summaries of the simulations.

## Declarations

Professor Mehanna is a National Institute for Health Research (NIHR) Senior Investigator. The views expressed in this article are those of the author(s) and not necessarily those of the NIHR, or the Department of Health and Social Care.
